# A Case of Rapidly Progressive Glomerulonephritis Associated With Metastatic Melanoma

**DOI:** 10.7759/cureus.73703

**Published:** 2024-11-14

**Authors:** Sitara Rao, Moeed Ahmed, Abdul R Ahmed, Julia Brown

**Affiliations:** 1 Department of Medicine, University of Illinois Chicago, Chicago, USA; 2 Department of Nephrology, Jesse Brown Department of Veterans Affairs Medical Center, Chicago, USA; 3 Department of Medicine, Northwestern University, Chicago, USA; 4 College of Medicine, Lahore Medical and Dental College, Lahore, PAK

**Keywords:** acute kidney injury, immune checkpoint inhibitor (ici), paraneoplastic syndrome, pauci-immune glomerulonephritis (gn), renal vasculitis

## Abstract

Pauci-immune glomerulonephritis is a rapidly progressive form of glomerulonephritis. It is distinguished from other rapidly progressive glomerular diseases by the lack of immune deposits seen on renal biopsy, hence the name “pauci-immune.” We present a case of pauci-immune glomerulonephritis in a patient with a history of malignancy that was treated with chemotherapy. Notably, the presentation of kidney injury coincided with the discovery of a new metastasis. Prompt treatment of glomerulonephritis is critically important to preserve renal function, though the optimal treatment strategy may not be straightforward in a patient with coexisting malignancy. Ultimately, this case illustrates that collaboration between patients, medical teams, and families is needed to optimize treatment strategy in unique circumstances.

## Introduction

Pauci-immune glomerulonephritis is a rapidly progressive and rare phenomenon. Most cases are associated with serum antineutrophilic cytoplasmic antibody (ANCA) positivity, though a minority are ANCA-negative [1]. Pauci-immune glomerulonephritis has previously been described in association with certain medications, infections, or neoplastic processes, though it can also be a primary process [2]. We present a case of seronegative pauci-immune glomerulonephritis to elucidate the appropriate steps of workup, as well as treatment considerations for this rare disease.

## Case presentation

An 88-year-old man presented to the emergency department with abdominal pain and progressive bilateral lower extremity and right upper extremity swelling, worsening over the past two weeks. Upon presentation, the patient was mildly hypertensive at 148/71. All other vitals were within normal limits. Labs on admission were significant for serum creatinine elevated to 1.6 from a baseline of around 0.9, blood urea nitrogen (BUN) of 45, albumin of 3.0, and urinalysis with 3+ blood and 3+ protein. The comprehensive metabolic panel (CMP) on admission is depicted in Table 1. CT chest/abdomen/pelvis showed mild to moderate ascites and anasarca and redemonstrated a known pancreatic head mass with ductal dilation that had been discovered during a recent hospitalization.

**Table 1:** Comprehensive metabolic panel upon presentation to the hospital An (L) in the result column denotes a measured value that is below the reference range, whereas an (H) in the result column denotes a measured value that is above the reference range.

Test name	Result	Reference range	Units
Sodium	142	136-145	mmol/L
Potassium	4.0	3.7-4.7	mmol/L
Chloride	101	98-109	mmol/L
CO_2_	30	20.0-31.0	mmol/L
Creatinine	1.60 (H)	0.67-1.17	mg/dL
Urea nitrogen	45 (H)	7-21	mg/dL
Glucose	105 (H)	70-99	mg/dL
Albumin	3.0 (L)	3.4-5.0	g/dL
Alkaline phosphatase	133 (H)	45-117	IU/L
Aspartate aminotransferase (AST)	18	10-37	IU/L
Alanine aminotransferase (ALT)	<9 (L)	10-65	IU/L
Calcium	7.8 (L)	8.7-10.4	mg/dL

The patient’s past medical history is significant for hypertension, coronary artery disease, heart failure with recovered ejection fraction, chronic obstructive pulmonary disease, hypothyroidism, thalassemia minor, low-grade papillary urothelial carcinoma now in remission, and stage 3 melanoma in remission with a new pancreatic head mass concerning for recurrence. He had no history of diabetes mellitus, nephrolithiasis, recurrent urinary tract infections, or recent nonsteroidal anti-inflammatory drug (NSAID) use. Three years prior to this presentation, the patient had undergone wide local excision for melanoma followed by one infusion of adjuvant pembrolizumab. Soon afterward, he was started on targeted immunotherapy with dabrafenib and trametinib. He could not tolerate this regimen due to excessive fatigue, anorexia, and weight loss. Nineteen days into the cycle, he was hospitalized for failure to thrive, and immunotherapy was stopped. PET-CT several months after stopping chemotherapy showed no evidence of active malignancy. The patient was subsequently lost to follow-up. His last dose of any immunotherapy was 31 months prior to the current presentation. Shortly before his current presentation, the patient was hospitalized for abdominal pain, and imaging revealed a new pancreatic head mass concerning for recurrence of melanoma. A biopsy of the lesion was performed 12 days prior to the current presentation, and results were pending.

Initial workup included urine studies that demonstrated nephrotic-range proteinuria, with an albumin-creatinine ratio of 3219 mg/g and a protein-creatinine ratio of 6205 mg/g. Nephrology was consulted, and nephrotic/nephritic syndrome workup was initiated, including HIV, syphilis, hepatitis B and C serologies, antinuclear antibody, vasculitis panel including ANCA, cryoglobulins, complement levels, serum protein electrophoresis, urine protein electrophoresis, and 24-hour urine collection. Besides mildly low C3 of 84.1 mg/dL (reference range: 90.0-170.0 mg/dL), the workup was entirely negative. Serum creatinine continued to rise rapidly, from 1.6 on presentation to around 4. A renal biopsy was performed, and while results were pending, the decision was made to start steroids empirically to attempt to prevent further renal damage.

**Figure 1:**
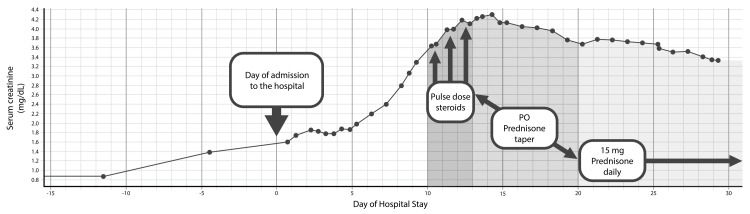
Trend of serum creatinine during hospital admission in relation to glucocorticoid administration

**Figure 2:**
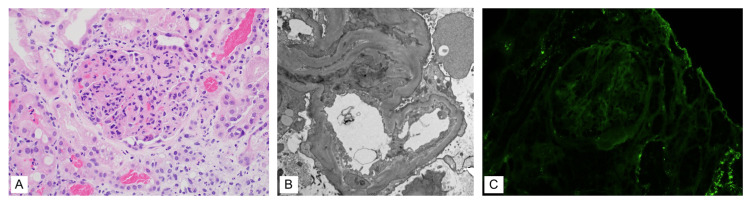
Representative histologic images from the patient’s renal biopsy Panel (A) depicts hematoxylin & eosin (H&E) stain at 400x magnification. Light microscopy reveals a proliferative glomerulonephritis with rare crescents combined with active tubulointerstitial nephritis. These findings are superimposed upon significant hypertensive nephropathy. Panel (B) is an electron microscopy image, magnification unknown. Electron microscopy shows signs of acute tubular injury with no electron-dense deposits or intracapillary fibrin tactoids seen. Panel (C) shows C3 immunofluorescence staining at 400x magnification. No immune deposits are visualized.

Creatinine reached a peak of 4.3 and then began a downtrend after three days of pulse-dose steroids. During this time, the results of the pancreatic lesion biopsy came back confirming metastatic melanoma. The oncology service recommended starting pembrolizumab, which required steroid dosing to be under 20 mg per day. Steroids were tapered to 15 mg daily, and pembrolizumab was started inpatient. Creatinine continued to downtrend on 15 mg oral prednisone daily, and proteinuria improved to 3 mg/g. A plot of serum creatinine during hospital admission is depicted in Figure 1 below. The renal biopsy came back showing pauci-immune glomerulonephritis with 2 out of 34 crescents, subtotal podocyte injury, acute tubulointerstitial nephritis, as well as arterio-/arteriolonephrosclerosis consistent with hypertensive nephropathy. Representative light microscopy (Figure 2A), electron microscopy (Figure 2B), and immunofluorescence (Figure 2C) histologic images from the renal biopsy are shown below. After completing one infusion of pembrolizumab inpatient, the patient was discharged to home on prednisone 15 mg daily with close outpatient follow-up.

## Discussion

Pauci-immune glomerulonephritis is a leading cause of rapidly progressive glomerulonephritis and is usually associated with ANCA positivity. However, up to 10% of cases are seronegative [1]. Standard treatment of pauci-immune glomerulonephritis involves immune suppression and modulation using agents such as glucocorticoids, azathioprine, mycophenolate mofetil, cyclophosphamide, and monoclonal antibodies such as rituximab.

Ronsin et al. conducted a case series that allowed them to develop a classification system of seronegative pauci-immune glomerulonephritis to better understand this rare phenomenon and its clinical spectrum. They classified the onset of seronegative pauci-immune glomerulonephritis after starting a new medication as a drug-induced phenomenon. They also described a separate group of cases that were associated with malignancy (either new malignancy or previously diagnosed and recently progressive) [2].

Regarding this case, it was postulated that the patient’s remote chemotherapy several years prior to his presentation may have played a role in his developing glomerulonephritis. Previous literature has described glomerulonephritis associated with immune checkpoint inhibitors (ICIs) as well as BRAF/MEK inhibitors used to treat melanoma [3], all of which the patient had received in the past. Pauci-immune glomerulonephritis and renal vasculitis are the most common types of glomerular lesions associated with ICI therapy [4]. A few cases of tubulointerstitial nephritis [5-7] and one case of nephrotic syndrome [8] have been reported after treatment with BRAF/MEK inhibitors dabrafenib and trametinib. We came across another case of glomerulonephritis after treatment with dabrafenib and trametinib and ICIs ipilimumab and nivolumab [9]. It is possible that this patient’s glomerulonephritis might have been a late presentation of chemotherapy complications, and further research should be done to elucidate the time course of renal complications following chemotherapy. However, this patient’s previous chemotherapy was very remote, so it is unlikely to be the cause of his renal presentation when another more likely explanation exists.

The concurrent discovery of a new metastasis suggests that the glomerulonephritis may be related to malignancy. We identified several reports describing presentations of seronegative pauci-immune glomerulonephritis in the setting of malignancy, including lung adenocarcinoma [10], colon carcinoma [11], and sarcoma [12]. In Ronsin et al.’s series, six patients were categorized as having malignancy-associated seronegative pauci-immune glomerulonephritis; of the six, three had a previously known malignancy that was in remission, as was the case with our patient. In all three of these patients, workup after the onset of seronegative pauci-immune vasculitis led to the discovery of cancer progression [2]. Given this patient’s onset of glomerulonephritis with concurrent discovery of a new metastasis, this case fits the clinical picture of malignancy-associated pauci-immune glomerulonephritis.

In this patient, the use of rituximab to treat the pauci-immune glomerulonephritis was considered. However, the potential drawbacks included a greater risk of sepsis and the possibility of reducing the efficacy of pembrolizumab therapy, which was being coordinated to treat the melanoma. To mitigate these risks, the hematology/oncology service preferred to delay rituximab administration for at least one week after pembrolizumab therapy. After pembrolizumab was given, the patient’s creatinine and proteinuria continued to improve. If the glomerulonephritis were cancer-related as suspected, it would be expected to respond to treatment of the cancer. Therefore, the discussion about rituximab was deferred to outpatient follow-up, and the patient was discharged to home.

## Conclusions

This case walks through a diagnostic approach for evaluating rapidly progressive glomerulonephritis. Notably, the presentation of seronegative pauci-immune glomerulonephritis in a patient with a history of cancer may reveal a progression of the malignancy. Clinicians must remain aware of such associations to ensure timely diagnosis and appropriate management.

This case also reinforces the necessity of an interdisciplinary approach when managing complex patients with dual diagnoses. In this situation, the oncology team, the nephrology team, and the patient and his family all worked together to define the goals of care and to establish an appropriate treatment plan. The collaboration of all parties was necessary to initiate effective cancer therapy and to tailor the treatment of glomerulonephritis.
